# The Long Non-Coding RNA GAS5 Cooperates with the Eukaryotic Translation Initiation Factor 4E to Regulate c-Myc Translation

**DOI:** 10.1371/journal.pone.0107016

**Published:** 2014-09-08

**Authors:** Guangzhen Hu, Zhenkun Lou, Mamta Gupta

**Affiliations:** Division of Hematology and Division of Oncology Research, Department of Internal Medicine, Mayo Clinic, Rochester, Minnesota, United States of America; Korea University, Republic of Korea

## Abstract

Long noncoding RNAs (lncRNAs) are important regulators of transcription; however, their involvement in protein translation is not well known. Here we explored whether the lncRNA GAS5 is associated with translation initiation machinery and regulates translation. GAS5 was enriched with eukaryotic translation initiation factor-4E (eIF4E) in an RNA-immunoprecipitation assay using lymphoma cell lines. We identified two RNA binding motifs within eIF4E protein and the deletion of each motif inhibited the binding of GAS5 with eIF4E. To confirm the role of GAS5 in translation regulation, GAS5 siRNA and *in vitro* transcribed GAS5 RNA were used to knock down or overexpress GAS5, respectively. GAS5 siRNA had no effect on global protein translation but did specifically increase c-Myc protein level without an effect on c-Myc mRNA. The mechanism of this increase in c-Myc protein was enhanced association of c-Myc mRNA with the polysome without any effect on protein stability. In contrast, overexpression of *in vitro* transcribed GAS5 RNA suppressed c-Myc protein without affecting c-Myc mRNA. Interestingly, GAS5 was found to be bound with c-Myc mRNA, suggesting that GAS5 regulates c-Myc translation through lncRNA-mRNA interaction. Our findings have uncovered a role of GAS5 lncRNA in translation regulation through its interactions with eIF4E and c-Myc mRNA.

## Introduction

Long noncoding RNAs (lncRNAs) are pervasive in the mammalian genome and are important in regulating a variety of biological functions through different molecular mechanisms [1]. Their role in transcription regulation has been comprehensively studied [2]. However, whether and how these lncRNAs are involved in translation regulation is still less known. Recently, ribosome-profiling studies revealed that many lncRNAs have similar ribosome occupancy as the translated regions of protein-coding genes [3] yet do not actually encode proteins [4], [5]. These reports suggest that the lncRNAs associated with the ribosome may serve a regulatory function.

Growth arrest-specific 5 (GAS5), a lncRNA critical to regulation of mammalian cell apoptosis and cell population growth, is frequently suppressed in many cancers [6], [7], [8], [9], [10]. Low GAS5 expression is associated with a poor prognosis in head and neck squamous cell carcinoma [11], and is considered to be a potential diagnostic marker and novel therapeutic target for non-small cell lung cancer [12]. GAS5 binds to the glucocorticoid receptor (GR) and acts as a decoy glucocorticoid response element (GRE), hence suppressing the up-regulation of gene expression by signaling through the GR [13]. For hematopoietic cells, GAS5 is essential to the normal cell growth arrest of both T-cell lines and non-transformed lymphocytes [14]. GAS5 is upregulated after rapamycin treatment, and is required in the inhibition of human T-cell proliferation by mammalian target of rapamycin (mTOR) antagonists [15], [16]. mTOR, a key component of mammalian TORC1 complex, is directly involved in protein translation, especially the protein translation of those mRNA with long and complex 5′ untranslated regions (UTRs) [17], [18]. Therefore, we speculated that GAS5 may be involved in the regulation of protein translation. Protein translation is tightly regulated at initiation step, which involves translation initiation complex eIF4F. eIF4F is a trimeric complex which is composed of eIF4G, eIF4E and eIF4A. We investigated the association of GAS5 with the eIF4F complex (particularly with eIF4E) and explored its role in the control of protein translation.

## Materials and Methods

### Cell lines

The lymphoma cell lines Jeko, Mino, Granta, and JVM2 were purchased from ATCC (Manassas, VA, USA). These cell lines were cultured in Roswell Park Memorial Institute medium supplemented with 10% fetal bovine serum. HEK-293T cell line was purchased from Open Biosystem (Huntsville, AL, USA) and was grown in the Dulbecco's Modified Eagle Medium supplemented with 10% FBS.

### Antibodies

Antibodies to c-Myc, Mcl-1, survivin, Bcl-2, eIF4E, eIF4G and RLP26 were from Cell Signaling Technology (Beverly, MA, USA). HA antibody was from abcam (Cambridge, MA USA). Actin antibody was purchased from Santa Cruz (Santa Cruz, CA, USA).

### Site directed mutagenesis and deletion

The plasmid HA-eIF4E in pcDNA3.1 (vector) was purchased from Addgene (Cambridge, MA, USA). The deletions of the RNA binding motifs and the mutations of 56W>A, 102W>A and 103E>A in the coding region of eIF4E were created with QuikChange II Site-Directed Mutagenesis Kits (Agilent, Santa Clara, CA, USA) according to the manufacturer's instruction and as described [19]. Briefly, the primers for point mutation or deletion were used for PCR with HA-eIF4E as template. Then the PCR products were treated with Dpn1 to degrade unwanted template plasmid and used for transformation of competent cells. Plasmids were then extracted from the clones and confirmed for mutation or deletion by DNA-sequencing. The following primers were used for the plasmids construction.

*HA-eIF4E^Del1^*: eIF4E^Del1^-F (5′ CCTATGTGGGAAGATGAGGACCTCGATCGCTTTTGG 3′) and eIF4E^Del1^-R (5′ CCAAAAGCGATCGAGGTCCTCATCTTCCCACATAGG 3′).

*HA-eIF4E^Del2^*: eIF4E^Del2^-F (5′ GATAGTGATTGGTTATCAGTTTGTTGTTTAACTCGAGC 3′) and eIF4E^Del2^-R (5′ GCTCGAGTTAAACAACAAACTGATAACCAATCACTATC 3′).

*HA-eIF4E^Del1&2^* was constructed based on *HA-eIF4E^Del1^* with primers eIF4E^Del2^-F and eIF4E^Del2^-R.

*HA-eIF4E^W56A^*: eIF4E^W56A^-F (5′-CTGGTTTTTTAAAAATGATAAAAGCAAAACTGCGCAAGCAAACCTGCGG-3′) and eIF4E^W56A^-R (5′-CCGCAGGTTTGCTTGCGCAGTTTTGCTTTTATCATTTTTAAAAAACCAG-3′).

*HA-eIF4E* ^WE102, 3AA^: eIF4E^WE102, 3AA^-F (5′- AGGATGGTATTGAGCCTATGGCGGCAGATGAGAAAAACAAACGGG-3′) and eIF4E^WE102, 3AA^-R (5′- CCCGTTTGTTTTTCTCATCTGCCGCCATAGGCTCAATACCATCCT-3′).

HA-eIF4E^Cap mutant^ was constructed by creating mutations 102W>A and 103E>A based on HA-eIF4E^W56A^ with primers eIF4E^WE102, 3AA^-F and eIF4E^WE102, 3AA^-R.

### Lentiviral stable transfection

eIF4E shRNA and non-silencing shRNA in pGIPZ vector were from Open Biosystem (Huntsville, AL, USA). Lentivirus carrying eIF4E or non-silencing shRNA was created with Trans-Lentiviral ORF Packaging Kit (Thermo Fisher Scientific, Waltham, MA, USA). Briefly, 6 million cells were plated into 100 mm plates overnight, and then the cells were transfected with pGIPZ-eIF4E and pGIPZ-non-silencing plasmids (9 µg of each). After 48 hours incubation, the supernatant was collected and the virus was purified by centrifugation at 3000 g for 15 minutes at 4°C. The virus was then incubated with 6 million HEK-293T cells in 100 mm plates for 48 hours, and the cells were selected with 2.5 µg/ml puromycin (Invitrogen, Grand Island, NY, USA) for 3 passages before the eIF4E protein level was checked by western blot.

### Transient plasmid transfection

For the RNP-immunoprecipitation experiment, 6 million HEK-293T cells were plated overnight. Cells were then transfected with 50 µg of plasmids and 125 µl of lipofectamine (Invitrogen, Grand Island, NY, USA) for 24 hours. For western blot, 0.7 million cells were plated overnight, and transfected with 10 µg of plasmids with lipofectamine. Transfected cells were harvested for western blot after 24 hours incubation.

### Transient siRNA transfection

For western blot and RNA extraction, 0.7 million cells were plated in 6-well plates overnight, then 100 pmol siRNA along with 10 µl lipofectamine were incubated with the cells for 48 hours. For [^3^H]-Leucine incorporation assay, cells were transfected with siRNA for 48 hours. After incubation cells were harvested and plated into 96-well-plate at a density of 10,000 cells/well and [^3^H]-Leucine incorporation assay was performed.

### [^3^H]-Leucine incorporation assay

HEK-293T cells were plated into 96-well-plate at a density of 10,000 cells/well overnight. Then 8 µL of 10 µCi [^3^H]-leucine was added into the cells and incubated overnight before the protein in the cells was precipitated with 10% trichloroacetic acid for 30 minutes. 6.1 µL of 3 M sodium hydroxide was added before the activity of [^3^H]-leucine was evaluated in 2450 microplate Counter (PerkinElmer, Waltham, MA, USA).

### Western blotting

0.7 million cells were plated into 6-well plates overnight, and treated with siRNA and plasmids as indicated; then collected and washed with ice-cold PBS. Western blot was performed after the cells were lysed with RIPA buffer as described previously [20]. Protein levels were normalized with Actin.

### Quantitative RT-PCR (Q-PCR)

RNA was extracted from 2 million cells with the RNeasy Mini Kit (QIAGEN, Germantown, MD, USA). cDNA was synthesized using 1–2 µg of total RNA with SuperScript III First-Strand Synthesis SuperMix (Invitrogen, Grand Island, NY, USA). Q-PCR was performed on the CFX96 real-time PCR detection system (Bio-Rad, Hercules, CA, USA). The program consisted 95°C for 15 minutes, then 45 cycles of 95°C for 10 seconds, 60°C for 30 seconds and 72°C for 30 seconds. Data analysis was performed by delta delta CT method with GAPDH as internal controls. The following primers with 1x EvaGreen (Biotum, Hayward, CA, USA) were used for quantifying mRNA levels of GAS5, eIF4E, c-Myc, Mcl-1, Bcl-2 and survivin.

*GAS5*: GAS5-F (5′ TGAAGTCCTAAAGAGCAAGCC 3′) and GAS5-R (5′ ACCAGGAGCAGAACCATTAAG 3′).

*c-Myc*: c-Myc-F (5′ GACGACGAGACCTTCATCAAAAAC 3′) and c-Myc-R (5′ AGGCCAGCTTCTCTGAGAC 3′).

*Mcl-1*: Mcl1-F (5′ CTGGGATTGAGAGGTTGATGAATG 3′) and Mcl1-R (5′ TGCCCAATCAGAGCCCATTATTTG 3′).

*survivin*: survivin-F (5′ AGGGTGGATTGTTACAGCTTCG 3′) and survivin-R (5′ CAGCAGTGTTTGAAATGACAGGC 3′).

*Bcl-2*: Bcl2-F (5′ GGAAACTTGACAGAGGATCATGC 3′) and Bcl2-R (5′ CGGATCTTTATTTCATGAGGCACG 3′).

*GAPDH*: GAPDH-F (5′ ATCACCATCTTCCAGGAGCG 3′) and GAPDH -R (5′ CAAATGAGCCCCAGCCTTC 3′).

*Actin*: Actin-F (5′ ACACCTTCTACAATGAGCTGC 3′) and Actin-R (5′ GAAGGTCTCAAACATGATCTGG 3′).

### RT-PCR

Primers used are shown in the above methods section. The program consisted of 95°C for 15 minutes, then 30 cycles of 95°C for 15 seconds, 60°C for 30 seconds and 72°C for 30 seconds, followed by 72°C for 10 minutes. The PCR products were then run on a 2% agarose (BioExpress, Kaysville, UT, USA) gel and photographed in an AlphaImager 3400 image detection system (San Leandro, CA, USA).

### Dual luciferase assay

Plasmid pRF was a kind gift from Dr. Gregory Goodall (Centre for Cancer Biology, at Adelaide, SA Australia). pRF-SL was constructed by the insertion of 4 stem loops between SV40 promoter and renilla luciferase. 0.25 million cells were treated with GAS5 or control siRNA for 48 hours in 24-well plate; then transfected with 50 ng pRF or pRF-SL plasmids by lipofectamine. Luciferase activity was measured with a dual luciferase assay kit (Promega, Madison, WI, USA). Renilla luciferase activity was normalized by firefly luciferase activity.

### RNP-immunoprecipitation (RNA-IP)

RNA-IP was performed with Magna RIP RNA-Binding Protein Immuno-precipitation Kit (Millipore, Billerica, MA, USA). Briefly, 80 million cells were lysed in 200µl RIP lysis buffer. Then the lysate was immuno-precipitated with eIF4E antibody or IgG along with protein magnetic beads. After proteinase K digestion, the RNAs pulled down with proteins were purified by phenol chloroform extraction and precipitated in ethanol. The RNAs were then re-suspended in 20 µl RNAse-free water and cDNA was synthesized with random primers using SuperScript III SuperMix (Invitrogen, Grand Island, NY, USA) and subjected to RT-PCR to detect GAS5 or GAPDH (internal control) transcripts. The RNA level was normalized with input (10%).

### Biotin pull-down assay

Biotin labeled DNAs were synthesized by Integrated DNA Technologies (Coralville, IA, USA). 20 million HEK-293T cells were lysed and incubated with 10 µg biotin-labeled DNAs overnight. The RNAs associated with biotin-labeled DNAs were then pulled down with Streptavidin Mag Sepharose (GE Healthcare, Madison, WI, USA) after a 1-hour incubation. RNAs was then washed and purified by phenol chloroform extraction. 20 µl RNAse-free water was used to re-suspend the RNA and 6 µl RNA was used for cDNA synthesis with random primers using SuperScript III SuperMix (Invitrogen, Grand Island, NY, USA).

### In vitro transcription

GAS5 DNA was amplified with primers of GAS5-cT-F (5′ TAATACGACTCACTATAGGGTTTCGAGGTAGGAGTCGAC 3′) and GAS5-cT-R (5′ TGGATTGCAAAAATTTATTAAAATTGGAGACACTG 3′). 200 ng of GAS5 PCR product was mixed with ATP, GTP, CTP and UTP along with T7 polymerase from MEGAscript T7 Kit (Invitrogen, Grand Island, NY, USA) and incubated 16 hours at 37°C. RNA was then treated with RNase-free DNase for 15 minutes at 37°C to remove the DNA template before the RNA was used for cell transfection. The product of the reaction without GAS5 templates was used as a vehicle control.

### Polysome analysis

Polysome analysis was performed as previously described [21]. Briefly, 20 million HEK-293T cells were treated with cycloheximide (CHX) (0.1 mg/ml) for 3 minutes and washed with ice-cold PBS containing CHX (0.1 mg/ml) for 3 times. Cells were then lysed in 400 µl of polysome extraction buffer (15 mM Tris-Cl, pH 7.4, 15 mM MgCl_2_, 0.3 M NaCl, 0.1 mg/ml CHX, 1 mg/ml heparin and 1% triton X-100). Then the cell lysate was centrifuged and the supernatant was loaded onto a sucrose gradient (10–50%) in Beckmann 9/16×3-3/4 mm polyclear centrifuge tubes. Fractions were collected after the centrifugation of 35,000 rpm at 4°C for 190 min. 2 µl of fractions was used to measure the absorbance at 254 nm to obtain the polysome profile, which was used to identify the polysome fractions and non-polysome fractions. RNA was then extracted with 8 M guanidine HCl and purified by lithium chloride precipitation and RNeasy Mini Kit (QIAGEN, Germantown, MD, USA). For Q-PCR, relative GAS5 mRNA abundance was calculated with delta CT method and normalized with total GAS5 mRNA in all the fractions. For western blot, 50 µl of each fraction was mixed with 2X loading buffer and samples were run on SDS gels. RLP26 (typically associated with polysome fractions) was used as a control.

### Statistics

The P value was calculated according to the two-tailed unpaired Student's t test. The quantification was processed with the software AlphaView SA (Alpha Innotech, San Leandro, CA).

## Results

### Association of GAS5 lncRNA with eIF4E

**Figure 1:**
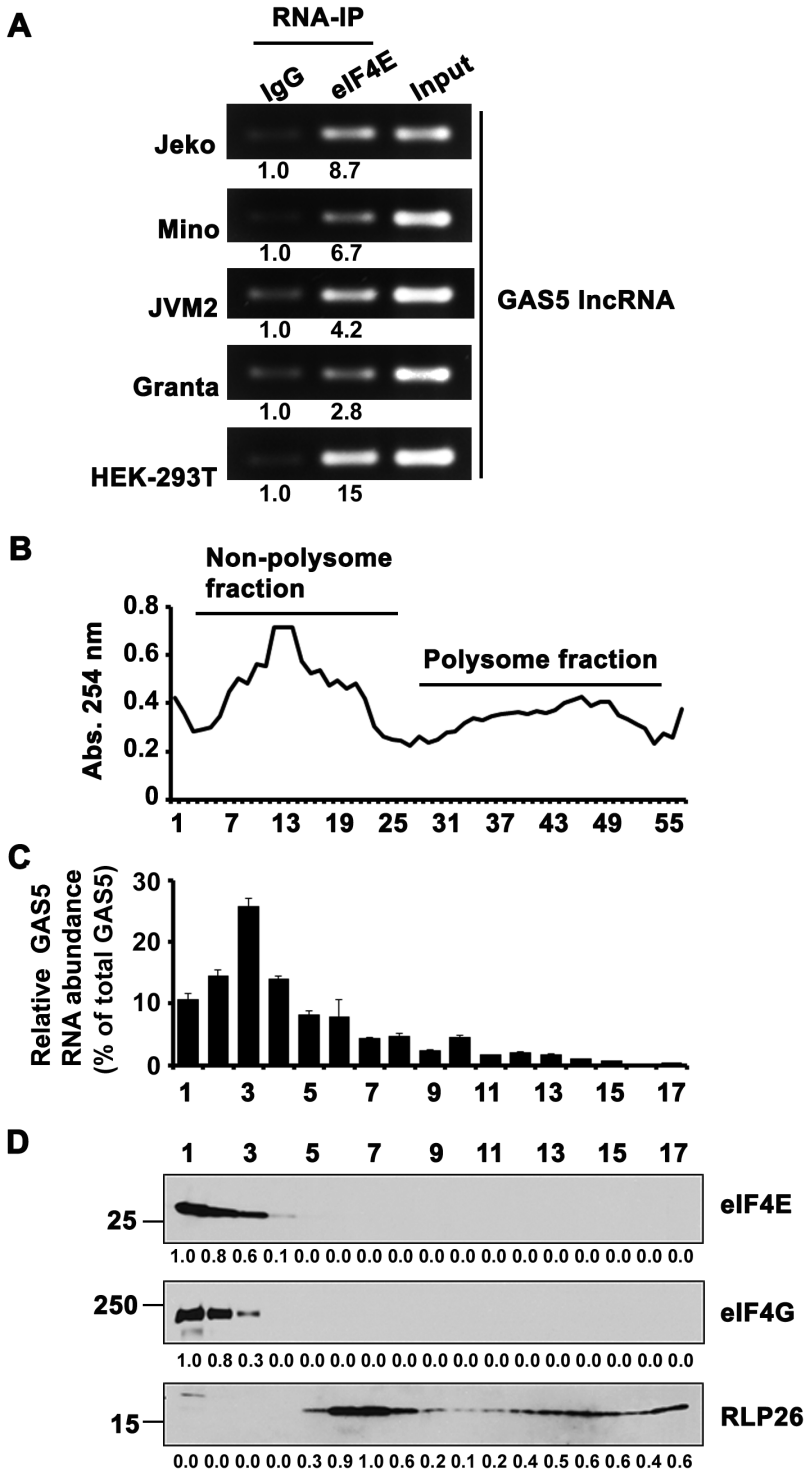
GAS5 interacts with eIF4E. (**A**) GAS5 mRNA was detected by RT-PCR after RNA-IP using eIF4E and IgG antibodies. The RNA-IP was repeated for two times with similar results. (**B**) The polysome and non-polysome fractions, as shown by the profile of the absorbance at 254 nm, were separated by sucrose gradient centrifugation in HEK-293T cells. The experiments were repeated three times. (**C**) The abundance of GAS5 lncRNA in the polysome and non-polysome fractions was measured by Q-PCR and normalized with total GAS5 mRNA in all the fractions. Bars represent mean ±SD from 3 replicates. Experiment was repeated three times, and a representative experiment is shown. (**D**) Protein levels of eIF4E and eIF4G in the polysome and non-polysome fractions were detected by western blot.

To explore whether GAS5 plays a role in regulating protein translation in lymphoma cells, we first detected the association of GAS5 with the translation initiation factor eIF4E since the initiation of translation is a key step in translation regulation [22]. RNA-IP results showed that GAS5 was enriched with eIF4E as compared with IgG control in all the lymphoma cell lines, Jeko, Mino, Granta and JVM2, as well as in HEK-293T cells **(** **Figure 1A** **)**. We also found that GAS5 was pulled down by eIF4G and eIF4A, which are two other components of eIF4F complex **(Figure S1)**. This confirmed that GAS5 is associated with translation initiation complex, eIF4F, suggesting its role in translation regulation.

To further confirm that GAS5 is associated with the active translation apparatus, we separated polysomes by sucrose gradient ultracentrifugation into the non-polysome fraction, where translation initiation occurs [23], and the polysome fraction, where mRNAs are associated with ribosomes and efficiently translated **(** **Figure 1B** **)**. GAS5 was primarily distributed in the non-polysome portion of the gradient as shown by the Q-PCR data **(** **Figure 1C** **)**. Moreover, eIF4E and eIF4G proteins were also found to be exclusively in the non-polysome fractions (**Figure 1D**). Collectively, these data demonstrate that GAS5 is associated with eIF4E at translation initiation step and may play a role in translation.

### Mechanism of GAS5 and eIF4E association

**Figure 2:**
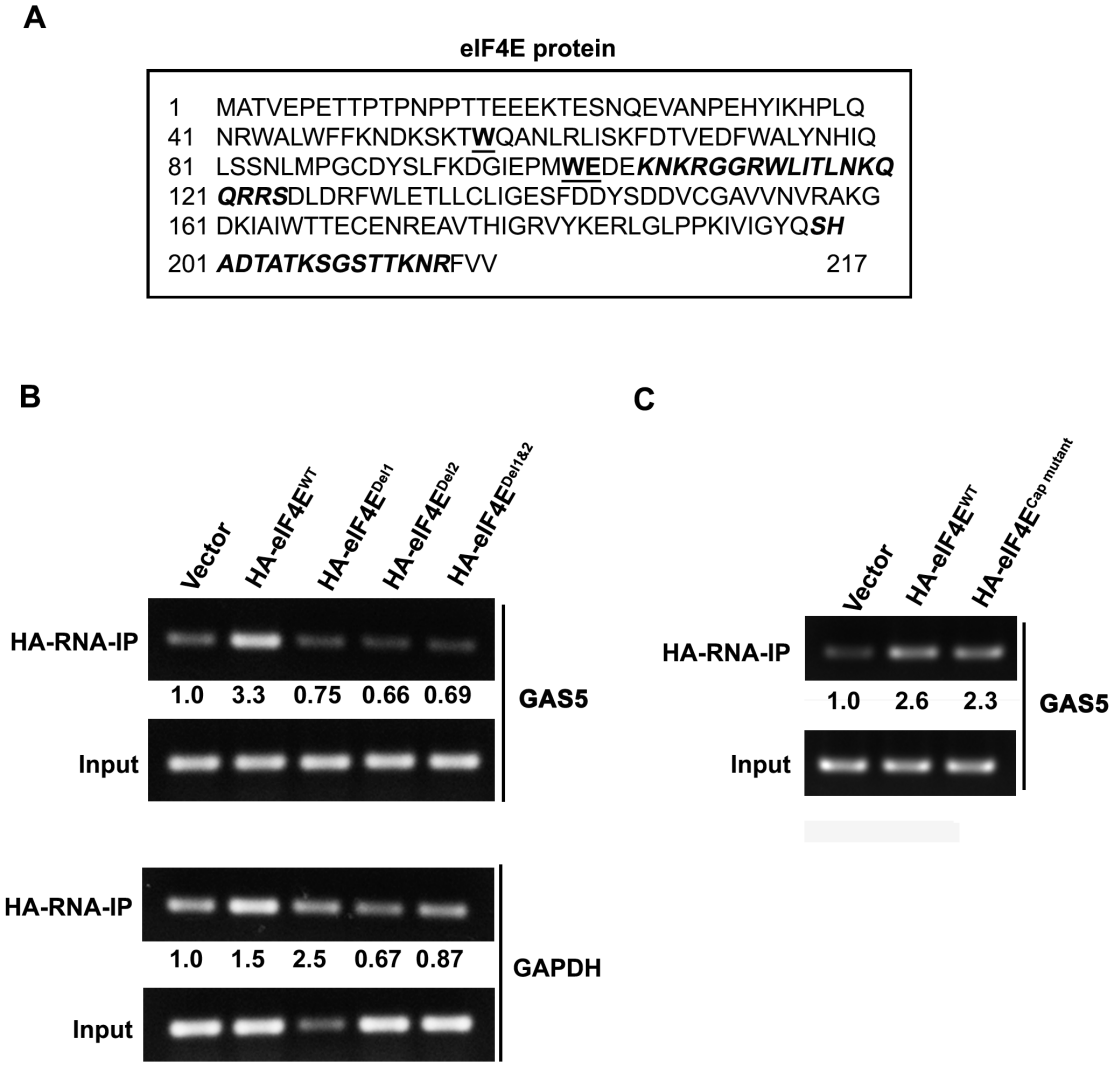
GAS5 binds to eIF4E through RNA binding motifs. (**A**) RNA binding motifs, which are italic and bold, in the eIF4E protein were predicted with 2 web-based tools, BindN and PPRInt. The motif, W56, W102 and E103 for m7G binding is bold and underlined. N-terminally located sequence is motif-1 and the C-terminal one is motif-2. (**B-C**) GAS5 RNA was detected by RT-PCR after RNA-IP assay using HA antibody in the cells transfected with (**B**) RNA binding deletion mutants (HA-eIF4E^Del1^, HA-eIF4E^Del2^ and HA-eIF4E^Del1&2)^ and (**C**) cap binding mutant (HA-eIF4E^cap mutant^). GAPDH was used as a control.

Because eIF4E binds with mRNA through its cap-binding motif, we postulated that GAS5 binds to eIF4E through motifs other than the cap-binding motif. To identify these motifs, the web-based tools, BindN and PPRInt, were used to predict RNA-interacting residues in eIF4E [24], [25]. Two RNA binding motifs were found based on these predictions **(** **Figure 2A** **)**. To test whether these two RNA binding motifs are actually involved in the binding of GAS5 to eIF4E we deleted RNA binding motif 1, motif 2, or both. Plasmids with the eIF4E wild type (wt) gene (HA-eIF4E^WT^) and eIF4E with RNA binding motifs deleted (HA-eIF4E^Del1^, HA-eIF4E^Del2^, HA-eIF4E^Del1&2^) were constructed and transfected transiently into HEK-293T cells. Unlike GAPDH mRNA the binding of GAS5 lncRNA to the wt eIF4E was increased by 3.3 fold as compared to vector alone, however deletions of either 1 or 2 or both RNA binding motifs abolished the binding of eIF4E to GAS5 **(** **Figure 2B** **)**. To check if the deletions affect the stability of eIF4E protein, the cells transfected with wt eIF4E and its deletion mutants were treated with CHX for various time points. Western blot data shows that the deletion of either motif did affect the stability of eIF4E (**Figure S2**).

In order to evaluate if the cap-binding motif is also involved in the binding of GAS5 to eIF4E, HEK-293 cells were transfected with a cap binding mutated plasmid (HA-eIF4E^cap mutant^). As expected, mutation of the cap-binding motif had no effect on the binding of GAS5 to eIF4E **(** **Figure 2C** **)**. Taken together, these data indicate that GAS5 binds to eIF4E through novel RNA binding motifs other than the cap-binding motif, suggesting a role of GAS5 in translation regulation.

### Effect of GAS5 inhibition and overexpression on global and specific mRNAs translation

**Figure 3:**
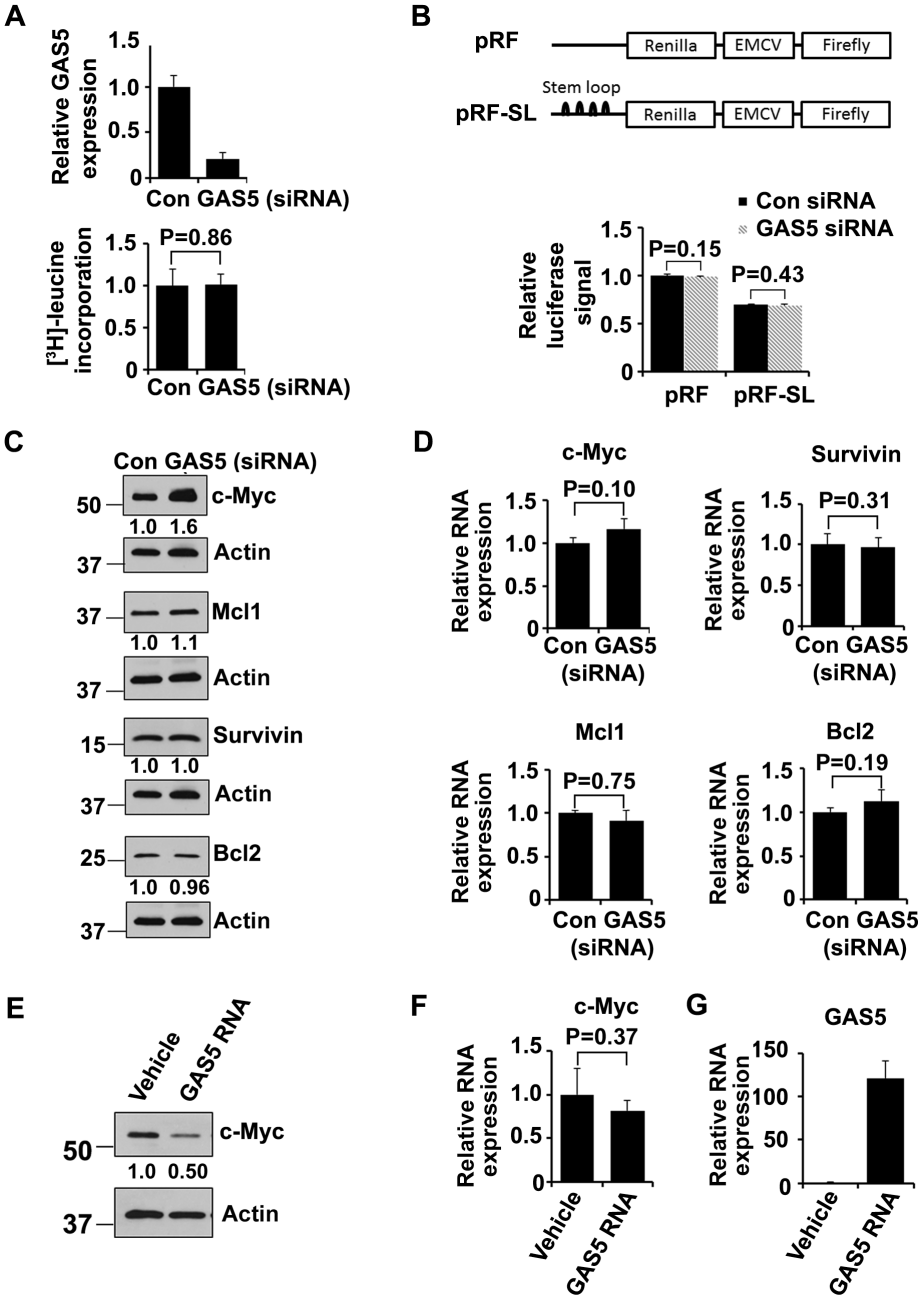
GAS5 suppresses c-Myc expression at protein level. (**A**) GAS5 was knocked down by siRNA in HEK-293T cells, and global protein translation was measured by [^3^H]-leucine incorporation assay. Bars represent mean ±SD from 3 replicates for Q-PCR and 6 replicates for [^3^H]-leucine incorporation assay. The experiments were performed two times. (**B**) pRF and pRF-SL plasmid were transfected into HEK-293T cells and cap-dependent protein translation efficiency was evaluated by luciferase assay. Bars represent mean ±SD from 4 replicates. The data were repeated three times. (**C**) The protein level of c-Myc (P = 0.007), Mcl1 (P = 0.88), survivin (P = 0.47) and Bcl2 (P = 0.47) was assessed by western blot (n = 3) after the cells were treated with GAS5 or control siRNA. (**D**) The mRNA level of c-Myc (P = 0.10), Mcl1 (P = 0.75), survivin (P = 0.31) and Bcl2 (P = 0.19) was quantified by Q-PCR after the cells were treated with GAS5 or control siRNA. Bars represent mean ±SD from 3 replicates. The experiment was repeated three times. (**E**) The protein level of c-Myc (P = 0.005) was assessed by western blot (n = 3) after the cells were transfected with *in vitro* transcribed GAS5 RNA. (**F-G**) The mRNA level of c-Myc (P = 0.37) (**F**) and GAS5 (**G**) was quantified by Q-PCR after the cells were transfected with *in vitro* transcribed GAS5 RNA. Bars represent mean ±SD from 3 replicates. The experiment was performed three times.

To determine the role of GAS5 in translation regulation, GAS5 was knocked down by siRNA and the effect on global protein translation was evaluated by a [^3^H]-leucine incorporation assay. Although 80% of GAS5 expression was blocked by the siRNA, this had no effect on global protein translation (**Figure 3A**). eIF4E controls translation of only those mRNAs with excessive secondary structure in their 5′ UTR [26]. We hypothesized that the GAS5 effect may be more specific to mRNA transcripts with complex 5′UTR structure. To test this hypothesis, we used bicistronic plasmids with renilla luciferase gene driven by SV40 promoter, which represent cap-dependent protein translation, and firefly luciferase gene controlled by EMCV, which represent cap-independent translation (pRF) [27] **(** **Figure 3B** **)**. Four stem loops were inserted between the SV40 promoter and renilla luciferase (pRF-SL) to block cap-dependent protein translation and thus mimic complex 5′UTR **(** **Figure 3B** **)**. The stem loops inhibited 30 percent of cap-dependent translation efficiency in both cells treated with control siRNA and GAS5 siRNA; however, GAS5 siRNA had no effect on the cap-dependent translation of both pRF and pRF-SL transfected cells **(** **Figure 3B** **)**. This indicated that the regulation of GAS5 on protein translation is not dependent on the complexity of the 5′UTR in mRNAs.

We then speculated that GAS5 is involved in regulation of specific mRNAs translation, especially the eIF4E downstream target genes *c-Myc, Mcl1, survivin, and Bcl2*. Indeed, c-Myc protein level was significantly increased (p = 0.007) when GAS5 was knocked down by siRNA; however, the protein levels of Mcl1, survivin, and Bcl2 were not affected **(** **Figure 3C** **)**. Moreover, the effect of GAS5 knockdown on c-Myc protein up-regulation was not due to transcriptional activation since mRNA level of Mcl-1, Bcl2, survivin along with c-Myc did not change significantly with GAS5 knocked down **(** **Figure 3D** **)**.

To further confirm the role of GAS5 on the specific regulation of c-Myc translation, *in vitro* transcribed GAS5 RNA was overexpressed in HEK-293 cells. Western blot data showed that enforced overexpression of GAS5 RNA significantly suppressed c-Myc protein level **(** **Figure 3E** **)**; however, c-Myc mRNA was not significantly affected as shown by the Q-PCR data **(** **Figure 3F** **)**. **Figure 3G** demonstrate successful GAS5 overexpression, which was 120 fold higher as compared to vehicle control. Taken together, our data showed that GAS5 specifically regulates c-Myc expression at the protein level without affecting global protein translation efficiency.

### GAS5 regulates c-Myc expression at translation level

**Figure 4:**
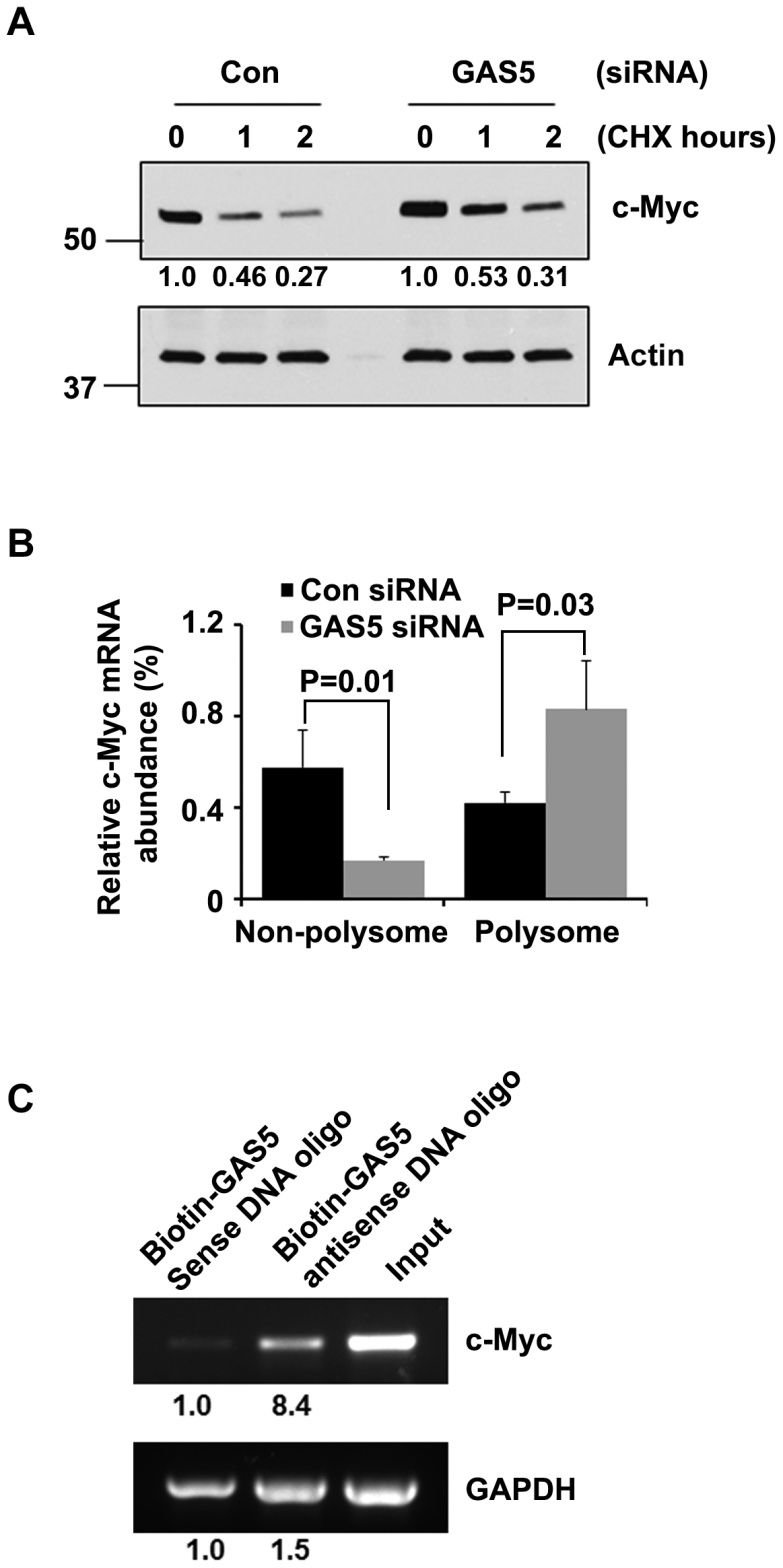
GAS5 suppresses c-Myc translation through direct binding with its mRNA. (**A**) The protein level of c-Myc was assessed by western blot after the cells were treated with GAS5 or control siRNA followed by the treatment of 100 µg/ml CHX for 1 and 2 hours. (**B**) c-Myc mRNA level associated with the polysome and non-polysome fractions of GAS5 and control siRNA transfected HEK-293T cells were evaluated by Q-PCR. Data was normalized with total mRNA (non-polysome + polysome). Bars represent mean ±SD from 3 replicates. (**C**) RNA from HEK-293T cells was pulled down by biotin-labeled GAS5 antisense DNA oligo with GAS5 sense DNA oligo as a control. The binding of c-Myc mRNA to GAS5 was evaluated by RT-PCR, in which GAPDH was used as a control.

c-Myc protein is typically unstable with a very short half-life in cells [28]. To examine whether the enhancement of GAS5 siRNA on c-Myc protein level was due to increased c-Myc protein stability, HEK-293T cells were treated with CHX after transfected with GAS5 or control siRNA. Western blot data showed that CHX treatment decreased c-Myc protein level in both control and GAS5 siRNA transfected cells at similar rates (**Figure 4A**). However, GAS5 siRNA enhanced c-Myc protein level as compared to control siRNA transfected cells without CHX treatment (**Figure 4A**).

To further clarify how GAS5 affects c-Myc translation; the polysome was extracted from cells transfected with GAS5 siRNA and control siRNA. The RNA of the polysome and non-polysome fractions was extracted and c-Myc mRNA level was evaluated by Q-PCR. **Figure 4B**, shows that polysome associated c-Myc mRNA level increased significantly (p = 0.03) in GAS5 siRNA transfected cells as compared with control siRNA transfected cells. c-Myc mRNA associated with non-polysome fractions decreased accordingly in the presence of GAS5 siRNA (p = 0.01) **(** **Figure 4B** **)**. These data indicate that GAS5 suppressed c-Myc translation by blocking the entrance of c-Myc mRNA into polysome. To confirm that GAS5 binds with c-Myc mRNA, biotin-labeled GAS5 antisense DNA oligo was used to pull down GAS5 and those RNAs bound with it. Biotin labeled GAS5 sense DNA oligo that is not able to pull down GAS5 was used as a control. To see if GAS5 specifically binds with c-Myc mRNA, GAPDH mRNA was used as a control for nonspecific binding [29]. As shown in **Figure 4C** **,** c-Myc mRNA but not GAPDH was enriched significantly with biotin-labeled antisense GAS5 DNA oligo as compared to the enrichment of GAPDH.

### Cooperation of eIF4E in GAS5 mediated c-Myc translation regulation

**Figure 5:**
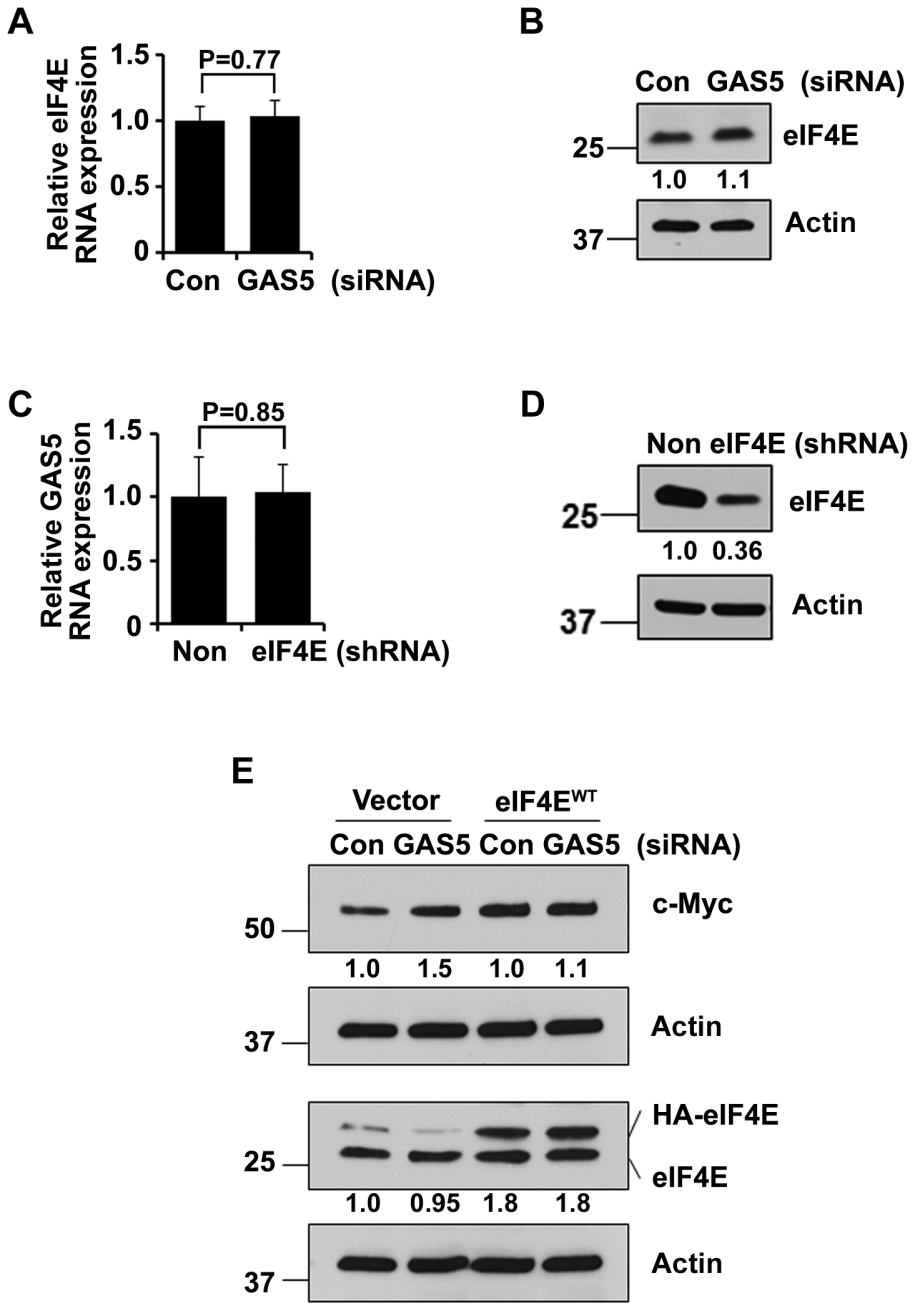
GAS5 cooperates with eIF4E to regulate c-Myc translation. (**A-B**) eIF4E expression at mRNA (**A**) and protein level (**B**) was assessed by Q-PCR and western blot after the cells were treated with GAS5 or control siRNA. Bars represent mean ±SD from 3 replicates. The experiment was performed three times. (**C**) GAS5 expression was quantified by Q-PCR in the HEK-293T cells transfected with non-silencing or eIF4E shRNA. Bars represent mean ±SD from 3 replicates. (**D**). eIF4E protein level after the cells were stably transfected with non-silencing or eIF4E shRNA. (**E**) The protein level of c-Myc and eIF4E was assessed after the cells were transfected with GAS5/control siRNA and eIF4E plasmid.

First we studied whether GAS5 inhibition would affect eIF4E expression. Interestingly, eIF4E expression was not much affected both at mRNA (**Figure 5A**) and protein levels (**Figure 5B**) in GAS5 inhibited cells. We also asked if eIF4E inhibition (eIF4E shRNA stable cells) would have any effect on GAS5 lncRNA expression. GAS5 expression was not affected when eIF4E expression was inhibited (**Figure 5C**). **Figure 5D** shows knockdown of eIF4E in eIF4E shRNA transfected HEK-293T cells. Next we asked if eIF4E is involved in the GAS5 mediated c-Myc translation regulation. We analyzed this by assessing the effect of GAS5 siRNA on c-Myc translation in eIF4E overexpressed HEK-293 cells. Interestingly, eIF4E overexpression itself increased the c-Myc protein expression, which was not further enhanced by GAS5 knockdown (**Figure 5E**). These results indicate that GAS5 cooperates with eIF4E in the regulation of c-Myc translation.

**Figure 6:**
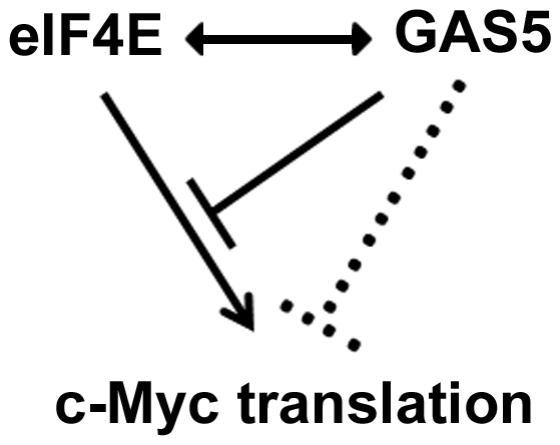
A schematic diagram of the GAS5 and c-Myc translation regulation.

Based upon our overall results, we propose that GAS5 suppresses c-Myc translation through interacting with eIF4E **(** **Figure 6** **)**.

## Discussion

c-Myc is a known transcription factor involved in several key biological processes including embryonic development and carcinogenesis [30], [31], [32]. We have recently shown that c-Myc is overexpressed in diffuse large B cell lymphoma and correlates with an inferior prognosis [33]. Like any other protein, expression of c-Myc is tightly controlled both at the transcription and translation level [34]. Protein translation in general can be controlled by several factors including micro RNAs [35], [36], RNA binding proteins, and RNA elements in mRNAs [37]. c-Myc protein translation has been shown to be regulated by RNA binding proteins [38], [39], [40] and structural elements within its mRNA [41], [42], [43]. Recently new mechanisms of protein translation control through lncRNAs have been described [21], [29], [44]. Our data presented here clearly demonstrate that c-Myc protein translation is negatively regulated through GAS5 lncRNA. Furthermore, we demonstrate that GAS5 suppressed c-Myc protein level without affecting its mRNA level or protein stability. This led us to hypothesize that GAS5 lncRNA may regulate c-Myc expression potentially at the level of translation. GAS5 lncRNA was shown to be upregulated in human T-cells in response to mTOR antagonists such as rapamycin [15], [16]. Consistent with a crucial role of mTOR in protein translation, these reports suggest a potential role of GAS5 in protein translation, which is clearly evident from our results presented here.

We have specifically shown that GAS5 lncRNA is recruited to the translation initiation complex, eIF4F, via direct binding with eIF4E, a key factor of translation initiation complex. A similar role of dendritic BC1 lncRNAs in regulating protein translation through eIF4A in neuron cells has been previously reported [45], [46], [47], [48], [49]. To our knowledge, this is the first report that GAS5 lncRNA binding to eIF4E protein requires RNA binding motif. Recently, lncRNAs have been shown to be bound with ribosome [3]. LncRNA-p21, which was found to inhibit translation of JunB and β-catenin by recruitment of translation repressor Rck, is mainly associated with polysome [29]. In contrast we have found that GAS5 is primarily associated with non-polysome fractions, suggesting its role in translation initiation regulation. In addition, another lncRNA antisense Uchl1, which activates uchl1 translation through the embedded SINEB2 repeat, also enhances the association of Uchl1 mRNA to active polysomes [44]. Distinct binding of GAS5 lncRNA to non-polysomal fractions therefore implies that the involved mechanism of c-Myc translation is probably different. Based on the fact that GAS5 interacts with translation initiation complex and is associated with monosome, it may likely decrease the efficiency of entrance of c-Myc mRNA into the polysome, but this need to be determined separately. Our results also showed that GAS5 interacts with c-Myc mRNA, which indicate how GAS5 targets specific mRNA. Like GAS5, the suppression of lincRNA-p21 on specific protein translation also involves interacting with its target mRNAs. Interestingly, another lncRNA treRNA, which suppress E-cadherin translation without direct interaction with the its mRNA [21]. In conclusion, we found for the first time that lncRNA GAS5 interacts and cooperates with eIF4E to regulate c-Myc translation, through lncRNA-mRNA interaction, thus broadening our understanding on the regulation of c-Myc translation.

## Supporting Information

Figure S1 **GAS5 interacts with translation initiation assembly.** GAS5 mRNA was detected by RT-PCR after RNA-IP using eIF4G, eIF4A, eIF4E antibody and IgG.
(TIF)

Figure S2 **The effect of the deletion of motif-1 and motif-2 on the stability of eIF4E protein.** **(A)** The protein level of eIF4E and its deletion mutants tagged with HA was assessed by western blot. **(B)** The protein level of eIF4E and its deletion mutants tagged with HA was assessed by western blot after the cells were treated with 100 µg/ml CHX for 0, 2, 4, 6, 8 and 24 hours.
(TIF)
