# Supplementary figures and images for: The Long Non-Coding RNA GAS5 Cooperates with the Eukaryotic Translation Initiation Factor 4E to Regulate c-Myc Translation
Source: PLoS One. 2014 Sep 8;9(9):e107016. doi: 10.1371/journal.pone.0107016 (PMC4157848; doi:10.1371/journal.pone.0107016)

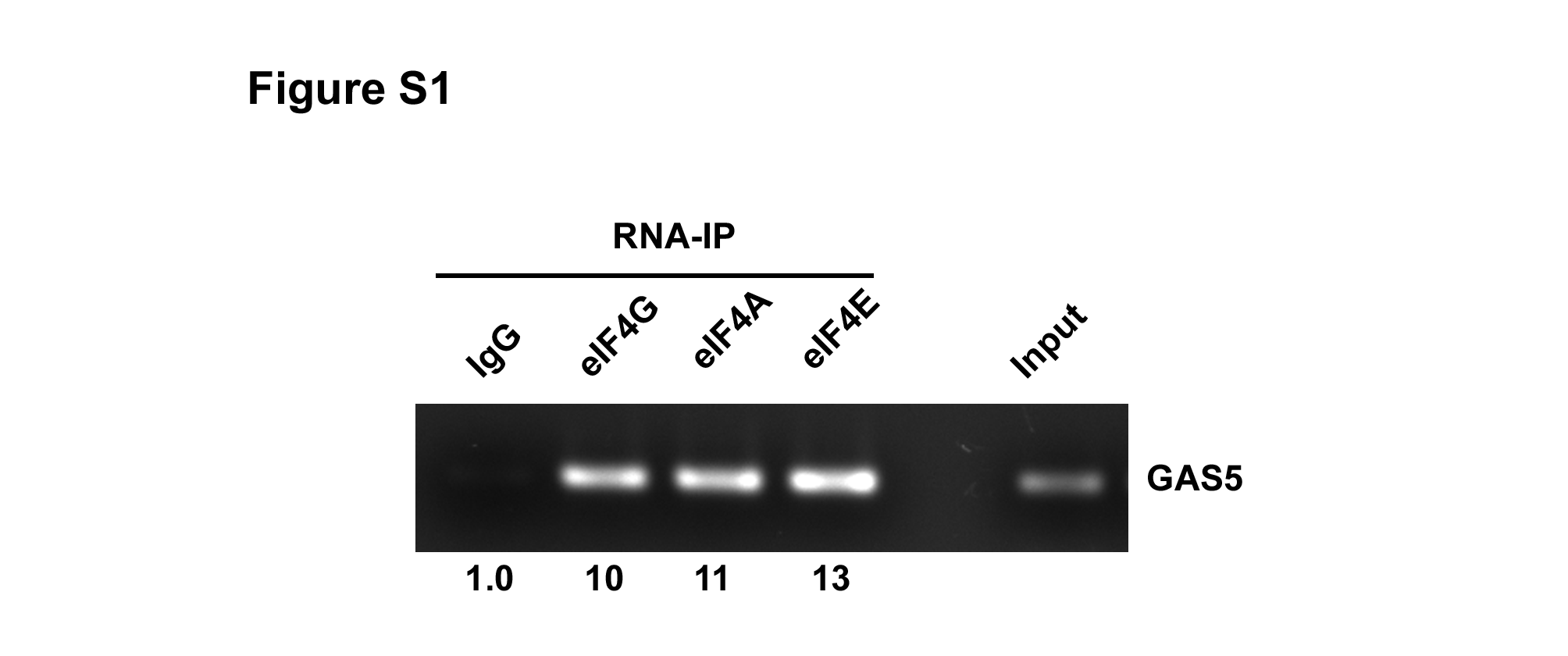

Supplement: Figure S1 — GAS5 interacts with translation initiation assembly. GAS5 mRNA was detected by RT-PCR after RNA-IP using eIF4G, eIF4A, eIF4E antibody and IgG. (TIF) [file pone.0107016.s001.tif]

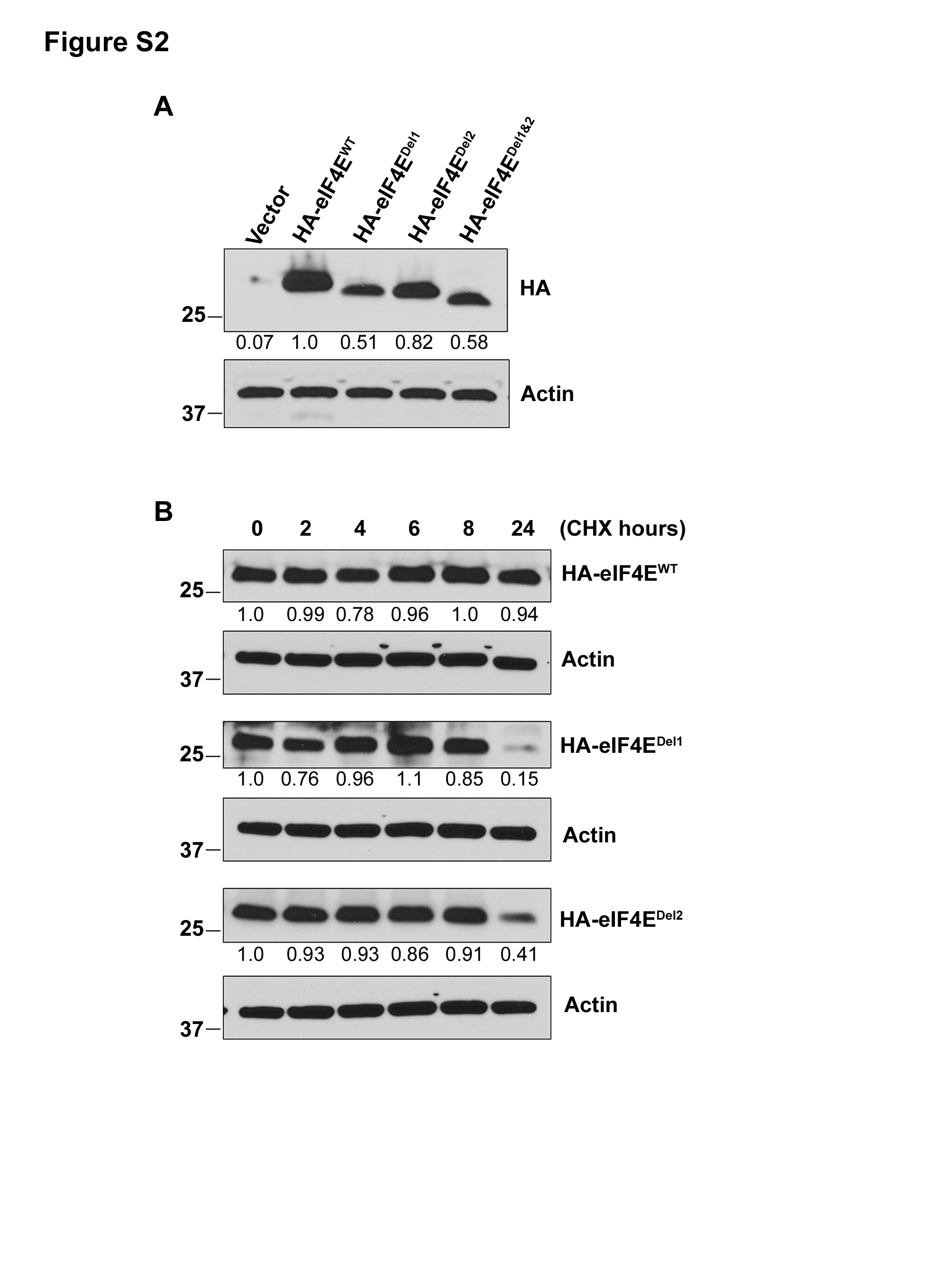

Supplement: Figure S2 — The effect of the deletion of motif-1 and motif-2 on the stability of eIF4E protein. (A) The protein level of eIF4E and its deletion mutants tagged with HA was assessed by western blot. (B) The protein level of eIF4E and its deletion mutants tagged with HA was assessed by western blot after the cells were treated with 100 µg/ml CHX for 0, 2, 4, 6, 8 and 24 hours. (TIF) [file pone.0107016.s002.tif]
